# Synthesis and Characterization of Functionalized Nanosilica for Zinc Ion Mitigation; Experimental and Computational Investigations

**DOI:** 10.3390/molecules25235534

**Published:** 2020-11-25

**Authors:** Zarshad Ali, Rashid Ahmad, W. Aslam Farooq, Aslam Khan, Adnan Ali Khan, Saira Bibi, Bushra Adalat, Mona A. Almutairi, Nafeesah Yaqub, Muhammad Atif

**Affiliations:** 1 Department of Chemistry, Hazara University, Mansehra 21300, Khyber Pakhtunkhwa, Pakistan; Zarshad11@yahoo.com (Z.A.); sairabushi@gmail.com (S.B.); bushraadalat@yahoo.com (B.A.); 2 Department of Chemistry, University of Malakand, Chakdara 18800, Dir Lower, Pakistan; rashmad@gmail.com (R.A.); aak95287@gmail.com (A.A.K.); 3 Chemistry Division, PINSTECH, PO Nilore 45650, Islamabad, Pakistan; jasminesi2007@gmail.com; 4 Department of Physics, College of Science, King Saud University, Riyadh 11451, Saudi Arabia; 436204157@student.ksu.edu.sa (M.A.A.); na-foo-sah@hotmail.com (N.Y.); muhatif@KSU.EDU.SA (M.A.)

**Keywords:** zinc adsorption, functionalized silica, adsorption models, surface modification, radiotracer, DFT analysis

## Abstract

Zinc is an essential trace metal and its concentration above 4ppm reduces the aesthetic value of water. This study explores the possibility of using functionalized nanohybrids as Zn(II) ion scavengers from aqueous solution. Functionalized nanohybrids were synthesized by the attachment of thiosemicarbazide to silica. The material was characterized by TGA, SEM, FTIR, EDX, and BET analysis, which revealed ligand bonding to silica. The functionalized silica was employed as Zn(II) ion extractant in batch experiments and removed about 94.5% of the Zn(II) ions at pH 7, near zero point charge (6.5) in 30 min. Kinetics investigations revealed that zinc adsorption follows an intra particle diffusion mechanism and first-order kinetics (K = 0.1020 min^−1^). The data were fitted to Freundlich, Dubinin–Radushkevich, and Langmuir models and useful ion exchange parameters were determined. The impact of co-existing ions on Zn(II) ion sequestration was also studied and it was found that the adsorbent can be used for selective removal of zinc with various ions in the matrix. Quantum mechanical investigations revealed that the Zn(II) ion adsorption on ZnBS1 is more favorable, having higher binding energy (BE) (−178.1 kcal/mol) and ∆*H* (−169.8), and making tridentate complex with the N and S sites of the chelating ligand. The negative ∆G and BE values suggest highly spontaneous Zn(II) adsorption on the modified silica even at low temperatures.

## 1. Introduction

Mining operations and widespread industrial applications of metals have led to an increased level of toxic heavy metals in the environment and is one of the most threatening issues of the modern world. The levels of metals in the environment [1,2] are higher than their recommended levels, which impose a severe threat to humans and other organisms [3]. The selective extraction of these metals from wastewaters, such as mining/industrial and seawater, has been a goal of researchers for the purpose of water purification, metallurgical extraction, and environmental remediation [4,5]. A number of solid-phase extractants have been employed for adsorption of heavy metals from waste and drinking water. These materials include clay [6], zeolite [7], activated carbons [8], ion exchangers [9], and polyurethane foam [10], etc. Some of them suffer from inherent limitations, such as low efficiencies, poor selectivity, and chemical and mechanical instabilities. To minimize these problems, the idea of chemical functionalization have gained popularity and a number of functionalized organic inorganic hybrids were prepared [11,12]. In this context, functionalized silica gained attention for use in many technological applications, such as sensors, separation, medicines, and catalysis. These porous adsorbents have high surface areas, tunable morphologies, and good chemical and mechanical stabilities. These materials have been widely used for water purification and have excellent adsorption capacities and selectivity [13,14].

Zinc is an essential metal for life as a co-factor for some enzyme and insulin production. Zinc and its compounds are of particular importance in steel coating, dry batteries, catalysts, plastics, ceramics, printing, and pharmacy and are also considered as a micro-nutrient for animals and plants. In surface and ground water, its level never exceeds 0.01 and 0.05 mg/L [15]. Above the permissible limit, zinc is harmful, due to its non-biodegradability and accumulation in the food chain and when its concentration exceeds 3 mg/L in water, it has a stringent taste, and hence, reduces its acceptability and aesthetic value [16]. Therefore, keeping the zinc concentration within acceptable limits in water is very much necessary [3]. This may be attained by conventional methods like chemical precipitation, membrane techniques, filtration, reverse osmosis, solvent extraction, and adsorption [17].

Although there are some reports of zinc extraction from aqueous solutions on functionalized silica [18,19,20], the zinc-retaining capability of these adsorbents was very low (0.0011–1.59 mmol/g). Furthermore, a great deal of adsorbents in the literature deal with the extraction of zinc in the presence of other co-existing metals and comprehensive reports of zinc uptake on the functionalized silica are relatively rare.

Keeping this and the economic importance of zinc in mind, we synthesized and characterized functionalized silica for the recovery of low-level zinc from solutions. The batch adsorption mode was employed for the quantitative determination of zinc in aqueous solutions. The effects of various parameters, such as the pH, adsorption time, zinc(II) concentration, temperature, and quantity of functionalized silica adsorbent, were explored in detail. The Zn(II) adsorption optimum conditions were determined and the results were modelled with adsorption models, such as Langmuir, Freundlich, and Dubinin–Radushkevich (D-R). The mechanism of adsorption responsible for the binding of Zn(II) to the adsorbent surface was explored and discussed. The kinetics of Zn(II) adsorption were exhaustively studied to investigate the nature of the diffusion and kinetic order of Zn(II) adsorption. The Zn(II) extraction was also studied in the presence of competing ions in the matrix to ascertain the selectivity of silica adsorbent for zinc extraction. Regeneration studies were also performed to ensure the recyclability of the adsorbent and reversible binding of Zn(II) to the adsorbent surface. Furthermore, the computational method was also used as a suitable tool for the investigation of the molecular properties of various systems and understanding the geometries, electronic structures, and coordination modes [18,19,20,21]. The binding modes of thiosemicarbazide ligand with Zn(II) ions using computational tools for the adsorption of Zn(II) ions on various sites of chelating ligand were also investigated by computing binding energies and thermodynamic parameters.

## 2. Materials and Methods

### 2.1. Chemical and Methods

The chemical used were of analytical grade and were used as obtained. Tetraethylorthosilicate 97% was used as the silica source and was acquired from Sigma Aldrich, St. Louis, MO, USA. Cetyltrimethylammonium bromide (CTAB) was used as the templating agent and was acquired from Sigma Aldrich. The linker 3-aminopropyltrimethoxy silane (3-APTMS) and sodium hydroxide (NaOH) were also provided by Sigma Aldrich, St. Louis, MO, USA. Thiosemicarbazide was provided by East Man (Dresden, Germany). The water used was de-ionized, distilled from potassium permanganate pyres still.

The Zn(II) radiotracer was prepared by neutron irradiation of pure zinc metal. In total, 20 mg of zinc metal were encapsulated in a tube and coated with aluminum. The metal was then exposed to a constant neutron activation of 5 × 10^3^ n·cm^−2^ s^−1^ of neutrons in a 10 Mw research reactor of PINSTECH Islamabad for 10 h. The irradiated sample was kept for one week to cool down. After cooling, the sample was dissolved in conc. HCl. Excessive acid was evaporated after repeated dilution with de-ionized water and heating to dryness. Finally, the radiotracer sample was diluted to 25 mL to make the stock solution. The radiotracer purity of the Zn(II) tracer was confirmed by a multichannel analyzer fitted with a germanium lithium detector (Canberra). Buffer solution of pH 1–8 was prepared by adding an appropriate volume of 0.1 molar HCl and KCl (pH 1–3), acetic acid/sodium acetate (pH 4–6), and boric acid/sodium hydroxide (7–8). pH was measured by a Metrohm 605 pH meter. A gross gamma detector (Tennelec, Canberra, Australia) equipped with an NaI(Tl) detector was used for measuring gamma counts.

### 2.2. Characterization Techniques

The morphological features of the synthesized silica adsorbents were examined by a scanning electron microscope, JSM 6490-A JEOL (Tokyo, Japan) at 20 kV. The powder silica samples were gold coated before the analysis. Energy dispersive spectroscopy (EDX) analysis was also carried out to investigate the elemental composition of the silica adsorbents.

Fourier transform infrared (FTIR) spectroscopy was used for the identification of the functional groups on the surface of the silica adsorbent. The silica powder samples were analyzed by an infrared spectrophotometer 100 of Perkin Elmer, having a resolution of 100 cm^−1^, using KBr pellets, and the samples were scanned in a wavelength range of 400 to 4000 cm^−1^, at a resolution of 4 cm^−1^. The % T (transmittance) was plotted against wavelength (cm^−1^) and the structural details were determined from the spectra.

Thermogravimetric analysis (TGA) was performed to investigate the thermal stabilities of the silica adsorbents. The weight losses for all the synthesized adsorbents were recorded by a TGA analyzer using a Pyris Diamond (Perkin Elmer, Norwalk, CT, USA). The thermograms of pure and functionalized silica adsorbents were recorded in a temperature range of 30 to 700 °C in nitrogen atmosphere at a rate of 10 °C/min.

### 2.3. Procedure of Zn(II) Adsorption

Zn(II) adsorption tests were conducted in batch mode at 26 ± 1 °C using pre-determined optimized conditions of 0.20 mg of functionalized silica, zinc ion concentration of 2.769 × 10^−4^ M, 30-min equilibration time, 5-min centrifuge time at pH 7 in a 5-mL volume. Zn % uptake was calculated by using Equation (1):

(1) $$\%\;\mathrm{Zn}\left(\mathrm{II}\right)\;\mathrm{adsorption}=\;\frac{A_{0}-A_{e}}{A_{0}}$$

where *A*_0_ and *A*ₑ represent the initial and equilibrium concentration of zinc, respectively. The distribution coefficient (*K*_D_) was determined by using Equation (2):

(2) $$K_{D}=\frac{\mathrm{quntity}\;\mathrm{of}\;\mathrm{Zn}\left(\mathrm{II}\right)\mathrm{adsorbed}}{\mathrm{quntity}\;\mathrm{of}\;\mathrm{Zn}\left(\mathrm{II}\right)\;\mathrm{in}\;\mathrm{solution}}\times \frac{\mathrm{solution}\;\mathrm{volume}\;\left(V\right)}{\mathrm{adsorbent}\;\mathrm{mass}\;\left(W\right)}=\left(\frac{\mathrm{mL}}{g}\right)$$

### 2.4. Synthesis of Grafted Silica

Silica and amine-functionalized silica nanospheres were prepared as reported earlier [22]. For synthesizing thiosemicarbazide silica, 5 g of ligand were suspended in 50 mL of de-ionized water in a flask and placed on a hot plate. In total, 30 mL of formaldehyde (37%) were added drop wise and refluxed for one hour, then 10 g of the pre-synthesized amine-functionalized silica were added to it and refluxed for half hour. The thiosemicarbazide silica was filtered and washed with distilled water, ethanol, and methanol to eliminate the un-reacted reagents and in a tube furnace at 50 °C for one hour.

### 2.5. DFT Simulations

The molecular simulations were performed on Gaussian 09 code [21] using B3LYP theory and 6–31 G (d,p) basis set for C, H, N, and S atoms by selecting LANL2DZ basis set for the Zn atom. The frequency calculations were performed using the same theory and basis set to obtain thermochemical parameters at 298.15 K and 1 atm to check whether the geometries were at local minima or not. The absence of an imaginary frequency confirmed that all the geometries were at local minima. Explicitly solvent effects (addition of water molecules) were used. The contribution of electrostatic effects to the binding energies and thermodynamic data were computed by employing the conductor-like polarizable continuum model (CPCM) [23,24]. The binding energies both in gas and solvent phases were computed by:

(3) $$BE=E_{\mathrm{Complex}}{}_{}-E_{(\mathrm{FG}+{{[\mathrm{Zn}(H}_{2}{O)}_{6}]}_{{}^{}}^{2+})}$$

where $E_{\mathrm{Complex}}$ is the total energy of the complex system (metal attached with FG) and $E_{(\mathrm{FG}+{{[\mathrm{Zn}(H}_{2}{O)}_{6}]}_{{}^{}}^{2+})}$ is the energy of the individual monomer (FG and [Zn(H_2_O)]^2+^). The change in enthalpy (∆*H*) and free energy (∆*H*) were also calculated:

(4) $$\Delta H_{ad}=H_{\mathrm{Complex}}-H_{(\mathrm{FG}+{{[\mathrm{Zn}(H}_{2}{O)}_{6}]}_{{}^{}}^{2+})}$$

(5) $$∆G_{ad}=∆H_{ad}\;-\;T∆S_{ad}$$

(6) $$∆G_{ad}=∆H_{ad}\;-\;T\left(S_{FG}+S_{(\mathrm{FG}+[\mathrm{Zn}\left(H_{2}O{)}_{6}{]}^{+2}\right)}\right)$$

where *H* is the total electronic and thermal enthalpy, *G* is the total electronic and thermal Gibbs free energy, and *S* is the entropy at 298.15 K and 1 atm.

## 3. Results and Discussion

### 3.1. Adsorbent Characterization

**Figure 1:**
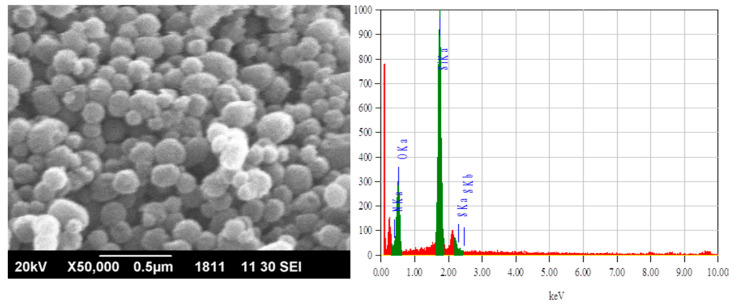
The SEM and EDX images of the thiosemicarbazide functionalized silica.

Textural properties of functionalized silica, such as pore volume, pore size, and pore diameter, were determined by the BET (Brunauer–Emmet–Teller) equation by the nitrogen gas sorption/desorption technique. The results indicated that the silica adsorbent was porous, having 920 m^2^/g surface area, 0.95 mL/g of pore volume, and 4.16 nm pore diameter. The SEM image and EDX analysis of the prepared sample are given in Figure 1. The EDX mapping showed oxygen, sulphur, and nitrogen moieties on silica.

**Figure 2:**
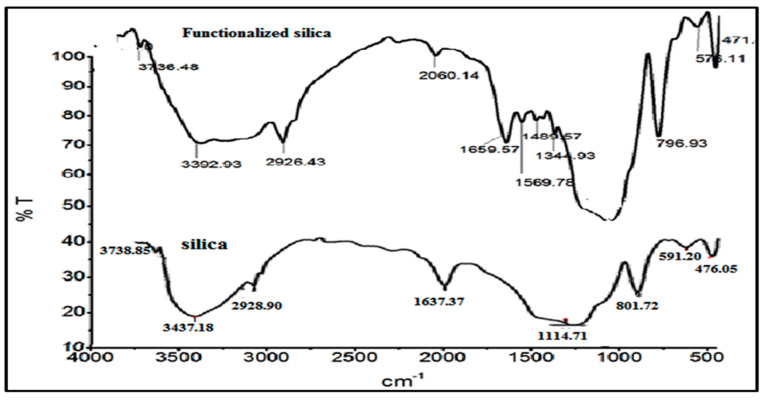
The FTIR analysis of the thiosemicarbazide functionalized silica.

The FTIR analysis of the silica and thiosemicarbazide functionalized silica is given in Figure 2. In the plain silica, a broad band can be seen at 3437 cm^−1^, which is due to the silanol (Si-OH) group skeletal vibration and physisorbed water molecules. The framework siloxane group (Si-O-Si) vibration occurred at 1114.0 cm^−1^. The stretching and bending vibrations (symmetric) of the siloxane groups appeared at 601.0 and 476.0 cm^−1^. After functionalization, some new groups appeared at 1344.0 and 1489.0 cm^−1^; these groups were ascribed to -N-H-C=S vibration [20]. The expected vibration of the -S=C- group at 730.0 to 1089 cm^−1^ was not present in our system, which might be overlapped with the strong skeletal vibration of the silica.

**Figure 3:**
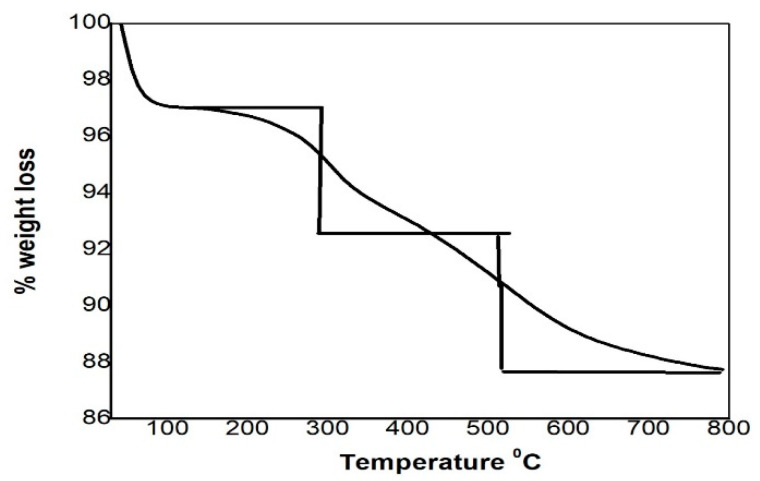
TGA analysis of the thiosemicarbazide functionalized silica.

The thermogravimetric (TGA) analysis of the synthesized functionalized silica is given in Figure 3. The functionalized silica lost its weight in three different regions. The first mass loss region was detected at a temperature of 30–250 °C, which is about 3% and is due to the elimination of physisorbed surface water [25]. The second mass loss was detected at the temperature of 250–500 °C owing to the decomposition of the organic functional group (-S=C-NH-) of the chelating ligand. The third mass loss was observed at 500–700 °C, and at this temperature, the silanols are condensed to siloxane groups on the surface [26].

### 3.2. Zn(II) Adsorption on Functionalized Silica

**Figure 4:**
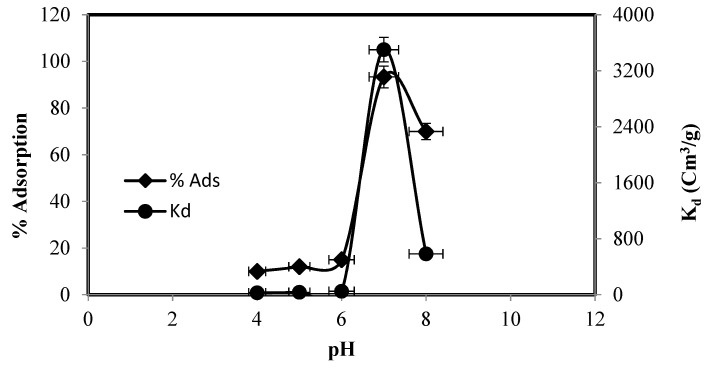
Effect of initial solution pH on the adsorption of Zn (II) and Kd values.

Medium pH has a key role in the removal of metal. It either protonates or deprotonates the functional groups and thus affects the functional group structure, metal speciation, and surface interactions [27]. The effect of pH on zinc adsorption and its distribution (K_d_ values) was examined in pH 1 to pH 8 and the outcomes are summarized in Figure 4. The Zn(II) adsorption varied with pH and in acidic medium (low pH), Zn(II) adsorption did not occur. Zinc adsorption started at pH 4 and attained maximum (94%) at pH 7 and then abruptly decreased.

With increasing pH, the following hydrolysis reactions of Zn^+2^ may occur in an aqueous system:

(7) $${\mathrm{Zn}}_{\left(\mathrm{aq}\right)}^{+2}+H_{2}O↔{\mathrm{ZnOH}}^{+}+H^{+},$$

(8) $${\mathrm{Zn}}_{\left(\mathrm{aq}\right)}^{+2}+2H_{2}O↔\mathrm{Zn}{\left(\mathrm{OH}\right)}_{2}+2H^{+},$$

(9) $${\mathrm{Zn}}_{\left(\mathrm{aq}\right)}^{+2}+3H_{2}O↔\mathrm{Zn}{\left(\mathrm{OH}\right)}_{3}^{-}+3H^{+}$$

**Figure 5:**
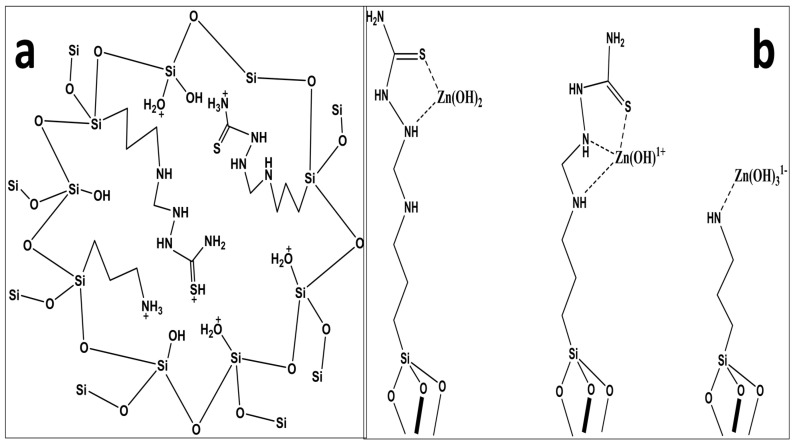
The possible protonation of the silica (**a**) and proposed mechanism for zinc adsorption (**b**).

At lower pH (˂6), zinc predominately exists as Zn^+2^, which coordinates to water molecules, and split to yield proton(s) and hydroxide species. The earlier researchers, on the basis of their metal speciation diagram, suggested that all the species of Zn^2+^ at pH 7.0 and below carry a positive charge either as Zn^2+^ or Zn(OH)^+^ [28] while, at higher pH, species like $\mathrm{Zn}{\left(\mathrm{OH}\right)}_{3}^{-}$ and precipitates of Zn(OH)_2_ may exist in solution [29]. The adsorption of zinc beyond pH 8 was not checked due to possible precipitation and hydrolysis of the metal ions in highly basic medium. The maximum adsorption of zinc at pH 7 may possibly be due to the presence of Lewis bases, such as NH_2_-, -NH, and -C=S, at pH 7. Lower zinc adsorption at lower pH may be attributed [30] to the protonation of the ligand functional groups owing to the abundance of the H^+^ in the medium, which could interact with the surface instead of the metal ion. pH 7 was chosen as a suitable medium for the extraction of zinc for other experiments. Figure 5 represents the possible protonation of the silica and the proposed mechanism for zinc adsorption.

**Figure 6:**
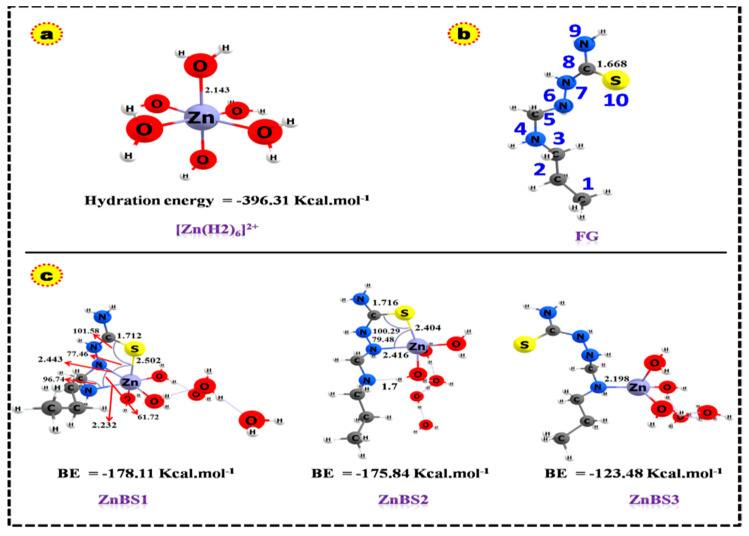
Optimized geometries of (**a**) hydrated [Zn(H_2_O)_6_]^2+^ (**b**) Functional group (**c**) complexes computed at the B3LYP level of theory and 6–31 G(d,p)/LANL2DZ basis set. All bond lengths in Å.

Based on the DFT simulation results, the minimized geometry of the functional group (FG), [Zn(H_2_O)_6_]^2+^, and Zn(II) binding with FG are depicted in Figure 6a–c while the optimized geometrical parameters and binding energies (BEs) are given in Table 1 and Table 2.

In aqueous media, Zn(II) is present in a hydrated form, which contains six water molecules in the first hydration shell [31]. The explicit solvent model, which considers waters in the first-hydration shell, in conjugation with the continuum salvation model, is the most commonly used model for the balance of the hydration shell [31]. Therefore, we modelled an interaction of hydrated Zn(II) ([Zn(H_2_O)_6_]^2+^) ion with three different sites of the function group, i.e., ZnBS1 (Zn(II) binding to 4N, 6N and 10S), ZnBS2 (Zn(II) binding to 6N and 10S), and ZnBS3 (Zn(II) binding to 4N), as shown in Figure 6c. In ZnBS1 and ZnBS2, the Zn(II) binds by two and three bonds with FG, making a one and two cyclic structure. The bond length of 4N—Zn in cycle 1 in ZnBS1 is 2.23 Å and the 5C—4N—Zn and 6N—Zn—4N angles are 96.7° and 61.7°, respectively. In cycle 2, the 6N—Zn(II) bond length is 2.44 Å, making (6N—Zn—10S) an angle of 77.5° while the 10S—Zn(II) bond length is 2.5 Å and the angle (8C—10S—Zn) is 101.5°. The C=S bond in ZnBS1 is stretched to 0.05 Å as compared to uncovered FG. The bond length in ZnBS2 of 6N—Zn is 2.42 Å, making an angle of 79.5° with in 6N and 10S atoms. Here, the C=S bond is stretched to 0.05 Å during the interaction of 10S with Zn(II) ion and the angle formed in 8C—10S—Zn is 101.58°. In ZnBS2, the 4N is not directly attached to the Zn(II) ion but is bonded through a hydrogen bond with the nearest water molecules (4N---HO), having a 1.7 Å bond length and making a 163° angle. The Zn(II) ion coordinated with FG through a coordinate covalent bond at ZnBS3 having a bond length of 2.20 Å (4N—Zn(II)) and a bond angle of 110.7°. The optimized geometrical analysis suggested that zinc adsorption is more feasible at ZnBS1 than ZnBS2 and ZnBS3, making three coordinated bonds with the electronegative sites (4N, 6N, and 10S).

**Figure 7:**
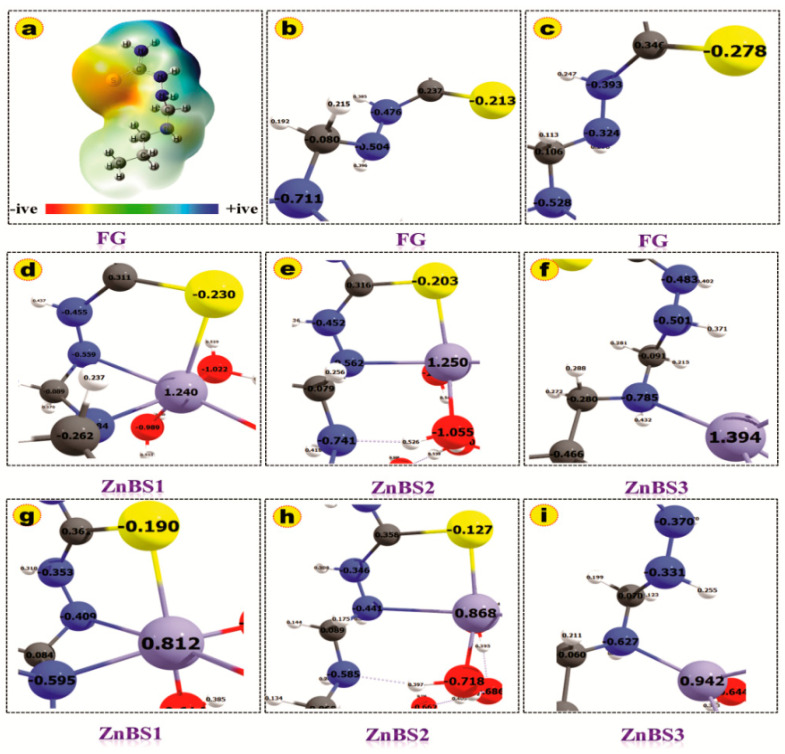
Computed (**a**) molecular electrostatic potential map; (**b**) NBO charges of FG; (**c**) Mulliken atomic charges of FG; (**d**) NBO charges of all complexes; (**e**) Mulliken atomic charges of all complexes. (**f**–**i**) Different modes of interactions of Zn(II) with functional groups. Only the coordinated zones are mentioned in this figure for clarity. All properties are computed in atomic unit.

The binding energies were computed for various coordination modes of hydrated Zn(II) ion with FG using the optimized geometries and values listed in Table 2. The BEs were computed as the difference of the total energy of the reactants and products. The more negative BE energies show the stronger metal ligand interactions and highly exothermic reactions [8,32]. The computed BEs for all the three binding sites are negative, which indicates that the ZN(II)–ligand interactions are highly exothermic. The binding energies in the gas phase are higher than the solvent phase, which may be due to the strong columbic interactions between the charged-Zn(II) cation and negative functional groups [33]. The order of BEs for various binding sites is ZnBS1 > ZnBS2 > ZnBS3. These results demonstrate that the FG has greater affinity for Zn(II) ions on ZnBS1 and ZnBS2 than ZnBS3. The Zn(II) ion makes three coordinate bonds with the FG in ZnBS1 as discussed above, which provide more stability to the complex and therefore has higher binding energy (BE = −178.11 Kcal/mol). The BE of ZnBS2 is not much lower (−175.8 Kcal/mol) than ZnBS1 (3 Kcal/mol lower), which is due to the two coordinate bonds of the Zn(II) ion with FG and one hydrogen bond. The additional hydrogen bond provided extra stability to the Zn(II) ion binding at ZnBS2, which is nearer to the ZnBS1 binding energy. While, on the other hand, the Zn(II) ion interaction in ZnBS3 is weaker than ZnBS1 and ZnBS2, having lower BE energy (−123.4 kcal/mol). The BE analysis established the fact that the more coordinated Zn(II) can extract the Zn(II) ions more efficiently. These results show good agreement with the previously reported work [8,34]. The hydration energy for [Zn(H_2_O)_6_]_2+_ is also listed in Table 2, which is also consistent with the reported literature [35], and the slight difference may be due to the use of a different basis set and level of theory selected. Figure 7a–i represents the molecular electrostatic potential map (MEP) of FG, natural atomic charges, and Mullikan atomic charges of FG and Zn(II) binding sites. The MEP map shows that the 4N, 6N, and 10S zones of FG are more electron-dense regions as depicted by the red color. The binding of Zn(II) ion is therefore more dominant on these sites. The blue region shows the electropositive region while the yellow and green zones represent the less negative and neutral regions, respectively. The variation of the Mullikan atomic charges and natural atomic charges is expressed on various atoms in Figure 7d–i. The variance in these charges clearly depicts the charge transfer phenomena during the complex formation between the Zn(II) ion and FG.

**Figure 8:**
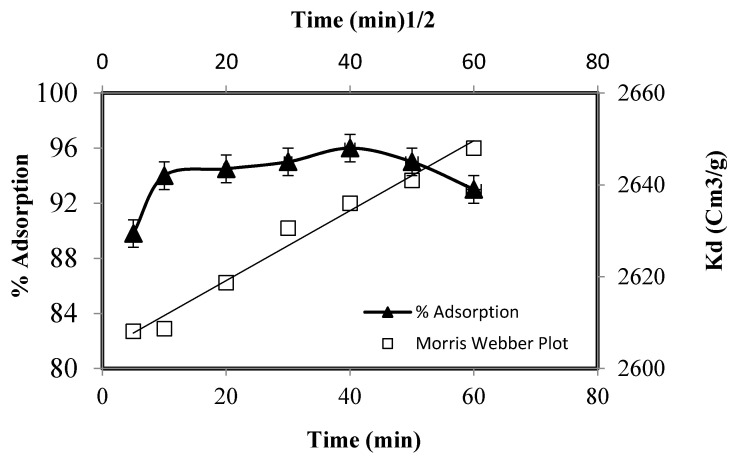
Adsorption of Zn(II) as a function of equilibrium time.

The kinetic investigations were performed by equilibrating 20.0 mg of the adsorbent, 2.769 × 10^−4^ mol/L of Zn (II) at optimized pH (pH 7) from 5–60 min, and the results are depicted in Figure 8. The adsorbent was recovered from the medium by centrifugation at different intervals and the solution was radio assayed for Zn(II) contents. In the beginning, the zinc uptake increased sharply (94%) up to 10 min of agitation and then increased slowly to attain a maximum of 96% at 40 min of agitation time. These investigations suggested that the removal of zinc by functionalized silica is fast and around 96% of the zinc is removed in the initial 40 min. The equilibration time is shorter, and the adsorbent has a good affinity for zinc, which could be removed quickly on its surface. The fast kinetics of zinc suggests that chemisorption may be the rate-limiting step [36].

The investigation of the mechanism and kinetics of adsorption is a necessary step for the application of adsorbent in industries. It has been suggested that the adsorption of metal on a solid surface is governed by film or intra particle diffusion or both of them [37]. Pertaining to the mechanism and kinetics of the zinc adsorption, the kinetic data were fitted to different rate and mass transfer equations. The Morris Webber equation was applied to investigate the film or particle diffusion nature of metal adsorption:

(10) $$q_{t}=R_{D}\sqrt{t,\;}$$

where *q_t_* is the amount of Zn(II) adsorbed at a given time and *R_D_* is the intra particle diffusion constant. Mousavi and co-workers [38] suggested that more than one adsorption steps is possible for zinc adsorption. The three possible steps are: diffusion of zinc from bulk solution onto the solid through the formation of a boundary layer, diffusion to the interior of the adsorbent, and/or Zn(II) adsorption on solid surface inside the pores. The final step is fast, and the adsorption of zinc is governed by the boundary layer or particle diffusion and decides the rate of zinc adsorption. Some researchers have suggested that formation of the boundary layer is the adsorption rate-determining (RDS) step [39]. The plot of *q_t_* v/s $\sqrt{t}$ did not pass through the origin, indicating that the rate-limiting step is not controlled by particle diffusion and the adsorption mechanism is complicated, which is controlled by two or more than two adsorption steps. The kinetic adsorption data were also fed to Reichenberg’s plot in the following form:

(11) $$F=\left(1-\frac{6}{\pi^{2\;}}e^{-\beta t}\right),$$

where $F=\frac{q_{t}}{q_{e}}$ is the ratio representing the Zn(II) concentration at time ‘*t*’ and *B_t_* is the mathematical function. The plot of βt/tv produced a straight line, which did not pass the origin, demonstrating that the sorption is chemically controlled. The Zn(II) adsorption data were also fed to the Lagergren first-order equation:

(12) $$\log\left(q_{e}-q_{t}\right)=\log q_{e}-\frac{Kt}{2.303},$$

where *K* is the first-order constant and *q_e_* and *q_t_* are the equilibrium and adsorbed concentration of Zn(II) on time ‘*t*’. The term $\log\left(q_{e}-q_{t}\right)$ when plotted v/s *t* gave a straight line, suggesting that the Zn(II) adsorption obeys the first-order equation. The value of *K* was 0.1020/min.

The effect of the Zn(II) concentration on its adsorption was studied over a six-times increase in concentration, using 20 mg of the functionalized adsorbent under the optimized conditions. The increase in the initial concentration increased Zn(II) adsorption sharply in the beginning and then became slower and attained a maximum of 94.46%. At this stage, the adsorbent surface became saturated with Zn(II) and a further increase in concentration did not increase the zinc adsorption, indicating that adsorbent saturation depends on the initial Zn(II) concentration [40]. Initially, the vacant adsorbent sites are easy to be approached by Zn(II) ions, but later on, they need to cross the energy barrier to diffuse into the solid surface [41]. To further explore the mechanism of the Zn(II) adsorption, Freundlich, Langmuir, and Dubinin–Radushkevich models were applied. The modelling of the adsorption data is necessary for technological applications. These models were applied in the linear form and the various parameters computed are tabulated in Table 3.

### 3.3. Zn(II) Adsorption in the Presence of Other Ions and Sorbent Regeneration

Wastewaters generally contain co-existing ions, which affect the removal of the desired metal. The assessment of these co-existing ions is necessary as they affect the removal of the target metal. In this study, various interfering anions and cations were added to the solution matrix and their impact on Zn(II) adsorption was explored. For this purpose, 10 mg of respective salt were added and its effect was investigated. The anions added, such as cyanide, oxalate, citrate, and sulphate, decreased the Zn(II) and some cations, such as mercury, silver, copper, and nickel, also decreased Zn(II) adsorption. On the other hand, cations, such as manganese, cadmium, lithium, iron, and EDTA, had no significant effect on zinc adsorption (Supplementary Materials). These findings revealed that this adsorbent can be selectively used for Zn(II) removal in competitive environments. The adsorbed zinc was completely recovered by treating it with 1 molar HCl. The adsorbent was regenerated by conditioning in the pH 7 solutions and reused for extraction of zinc. Functionalized adsorbent was stable after three sorption/desorption cycles and its adsorption capacity was not altered significantly. The adsorption efficiency of the regenerated sorbent was very closer to the freshly prepared (94.5%) sorbent up to three regenerations cycles, i.e., 94.1%, 94%, and 94.3%, respectively.

### 3.4. DFT-Based Thermodynamic Studies

The thermodynamic study provides additional information, which helps in understanding the adsorption process [42]. In thermodynamic studies, researchers have mostly analyzed the change in enthalpy, free energy, and entropy. These thermodynamic parameters assist in the practical applications of the adsorption mechanism [43]. In the present study, we computed the thermodynamic parameters using the same level of theory and basis sets and the values are reported in Table 2. The negative ∆*H* described the exothermic Zn(II) adsorption. Furthermore, if the ∆H values for Zn(II) ion adsorption are more than 10 Kcal/mol, the adsorption is chemical and vice versa [42,43]. The computed values of ∆*H* for all different sites are highly negative, which proved the exothermic and chemisorption process. The values of the calculated ∆S are negative, describing the lesser disorder at the solid–liquid interface. The tabulated values of ∆G are negative for all binding sites, which evidenced the feasible and spontaneous Zn(II) adsorption. The more negative ∆G suggested the more negative BE, which showed higher Zn(II) adsorption on the modified silica surface and that the reaction is spontaneous at low temperatures.

## 4. Conclusions

This study reported the fabrication of functionalized silica adsorbent and its successful application for the extraction of zinc. The adsorbent was used for Zn(II) extraction in batch operations using the radiotracer technique. The adsorbent can remove about 94.5% of Zn(II) ions at pH = 7, near zero point charge (pHzpc = 6.5) of the adsorbent in 40 min of equilibration. The zinc adsorption is dominated by particle diffusion and follows the first-order equation with *K* = 0.1020/min. The Freundlich, Langmuir, and D-R isotherms were followed over a good range of Zn(II) concentration. The adsorbent can be selectively used for zinc extraction in the presence of other ions. The Zn(II) adsorbed can be recovered and the adsorbent can be reconditioned by treatment with 1.0 M HCl solution. In addition, DFT analysis showed that the zinc is greatly adsorbed on the silica surface by making strong coordinated bonds with N and S atoms of functional groups. The binding energy value for ZnBS1 is higher (−178.1 Kcal/mol), suggesting the stronger adsorption of Zn(II) ions. The higher negative ∆*H* values of Zn(II) ion adsorption indicate the exothermic and chemisorption process. The negative ∆*G* values confirmed that the adsorption of Zn(II) ions on functionalized silica is a spontaneous process.

## Figures and Tables

**Table 1:** Optimized geometrical parameters bond lengths (Å) and angles (°) at the B3LYP/6–31 G(d,p)/LANL2DZ method.

**Specie**	**Bond Lengths (Å)**					
	**C=S**	**Zn—O**	**4N—Zn**	**6N—Zn**	**10S—Zn**	**4N…HO**
FG	1.67					
FG-Zn-1	1.71		2.23	2.44	2.50	
FG-Zn-2	1.72			2.42	2.40	1.7
FG-Zn-3			2.20			
[Zn(H2O)6]^2+^		2.14				
**Specie**	**Bond Angles (°)**					
	**5C—4N—Zn**	**4N—Zn—6N**	**6N—Zn—10S**	**8C—10S—Zn**	**5C—4N—Zn**	
FG-Zn-1	96.74	61.72	77.46	101.58		
FG-Zn-2			79.48	100.29		
FG-Zn-3					110.7	

**Table 2:** Computed thermodynamic parameters at 298.51 K and 1 atm $\left(∆E_{BE},∆H_{BE},∆G_{BE}\;\mathrm{and}\;∆S_{BE}\;\mathrm{Kcal}\cdot {\mathrm{mol}}^{-1}\right)$ using B3LYP theory and 6–31 G(d,p)/LANL2DZ basis sets.

Complex	$\Delta E_{BE}$		$\Delta H_{BE}$		$\Delta G_{BE}$		$\Delta S_{BE}$	
	Gas Phase	Solvent Phase	Gas Phase	Solvent Phase	Gas Phase	Solvent Phase	Gas Phase	Solvent Phase
Zn(H_2_O)_6_	−396.3	−80.91	−394.37	−78.91	−362.61	−69.43	−218.23	−65.31
ZnBS1	−178.11	−59.89	−169.82	−57.45	−140.76	−49.81	−97.46	−51.31
ZnBS2	−175.84	−58.90	−168.35	−54.71	−140.84	−45.72	−92.25	−49.10
ZnBS3	−123.48	−41.37	−117.77	−40.3	−86.66	−37.6	−104.34	−54.4

**Table 3:** Adsorption model constants for Zn(II).

Isotherm Model	Constants	Values
Langmuir isotherm	Q (µmol g^−1^)	20,000
	b × 10^3^ (L mol^−1^)	3.8
	R^2^	0.99
Freundlich isotherm	1/n	0.1627
	Cm (mmol g^−1^)	1.18
	R^2^	0.64
D-R isotherm	β (KJ^2^ mol^−2^)	−0.0015
	Xm (mmol g^−1^)	27.20
	Es (KJ mol^−1^)	18.25
	R^2^	0.97

## Supplementary Materials

The following are available online at Table S1: Effect of anions on the sorption of Zn(II) (2.769 × 10^−4^ M) on to TCS functionalized nano silica, silica (20 mg) after 30 min agitation time and pH 7.0, Table S2: Effect of cations on the sorption of Zn(II) (2.769 × 10^−4^ M) on to TCS functionalized nano silica, (20 mg), agitation time 30 min and pH 7.0.
