# Supplementary material for: Synthesis and Characterization of Functionalized Nanosilica for Zinc Ion Mitigation; Experimental and Computational Investigations
Source: Molecules. 2020 Nov 25;25(23):5534. doi: 10.3390/molecules25235534 (PMC7728340; doi:10.3390/molecules25235534)
Supplement: Supplementary file 1 [file molecules-25-05534-s001.pdf]

Table S1. Effect of anions on the sorption of Zn (II) (2.769×10^{-4} M) on to TCS functionalized nano silica, silica (20 mg) after 30 min agitation time and pH 7.0.

Compound	Interfering ion	Sorption (%)
Blank	Nil	95
$K_2Cr_2O_7$	$Cr_2O_7^{-2}$	95
$Na_2B_4O_7$	$B_4O_7^{-2}$	95
$NaNO_3$	NO_3^{-1}	93
KF	F^{-1}	93
KI	I^-	93
$Na_2S_2O_3$	$S_2O_3^{-2}$	87
$Na_2C_2O_4$	$C_2O_4^{-2}$	44
$C_6H_5O_7Na_3$	$C_6H_5O_7^{-3}$	36
KCN	CN^{-1}	1

Table S2. Effect of cations on the sorption of Zn(II) (2.769×10^{-4} M) on to TCS functionalized nano silica, (20 mg), agitation time 30 min and pH 7.0.

Compound	Interfering ion	Sorption (%)
Blank	Nil	95
EDTA	EDTA	93
Fe $(NO_3)_3$	Fe^{+3}	93
$CdCl_2$	Cd^{+2}	86
Li Cl	Li^{+1}	82
$MnCl_2$	Mn^{+2}	82
$AgNO_3$	Ag^+	55
Cu SO_4	Cu^{+2}	11
$Ni(NO_3)_2$	Ni^{+2}	6
Hg ($NO_3)_2$	Hg^{+2}	3
